# Sensory-to-Motor Overflow: Cooling Foot Soles Impedes Squat Jump Performance

**DOI:** 10.3389/fnhum.2020.549880

**Published:** 2020-10-09

**Authors:** Mia Caminita, Gina L. Garcia, Hyun Joon Kwon, Ross H. Miller, Jae Kun Shim

**Affiliations:** ^1^Department of Kinesiology, University of Maryland, College Park, MD, United States; ^2^Neuroscience and Cognitive Science Program, University of Maryland, College Park, MD, United States; ^3^Fischell Department of Bioengineering, University of Maryland, College Park, MD, United States; ^4^Department of Mechanical Engineering, Kyung Hee University, Yongin-Si, South Korea

**Keywords:** cutaneous feedback, squat jump height, sensorimotor integration, maximum force production, motor cortex

## Abstract

Evidence from recent studies on animals and humans suggest that neural overflow from the primary sensory cortex (S1) to the primary motor cortex (M1) may play a critical role in motor control. However, it is unclear if whole-body maximal motor tasks are also governed by this mechanism. Maximum vertical squat jumps were performed by 15 young adults before cooling, then immediately following a 15-min cooling period using an ice-water bath for the foot soles, and finally immediately following a 15-min period of natural recovery from cooling. Jump heights were, on average, 3.1 cm lower immediately following cooling compared to before cooling (*p* = 3.39 × 10^−8^) and 1.9 cm lower following natural recovery from cooling (*p* = 0.00124). The average vertical ground reaction force (vGRF) was also lower by 78.2 N in the condition immediately following cooling compared to before cooling (*p* = 8.1 × 10^−5^) and 56.7N lower following natural recovery from cooling (*p* = 0.0043). The current study supports the S1-to-M1 overflow mechanism in a whole-body dynamic jump.

## Introduction

The understanding of sensory processing and motor outputs in the central nervous system (CNS) has long been a vast topic of study. During the mid 20th century it was discovered that sensory and motor function occupy distinct spatial locations in the cerebral cortex, with the mapping often referred to as the sensory and motor homunculi, respectively (Penfield and Boldrey, 1937; Penfield and Rasmussen, 1950). At the end of the 20th century, the somatotopic organization of the motor cortex hand area was more closely examined and demonstrated evidence of spatial overlap of cortical territories for movement of different finger digits in non-human primates (Schieber and Hibbard, 1993). Recently, rodent studies have shown neuroanatomical connections between the designated “sensory” and “motor” areas of the brain. The primary somatosensory barrel cortex has been shown to form a direct and prominent motor control pathway, similar to that originating from the motor cortex, in mice (Matyas et al., 2010). Overlapping sensory and motor representations have also been observed in the rodent hind limb (Donoghue and Wise, 1982). More recent studies have provided experimental evidence for the projection of primary somatosensory cortex (S1) to the primary motor cortex (M1: Rocco-Donovan et al., 2011; Petrof et al., 2015). These studies have provided evidence of inputs from S1 to M1 and the possibility of modulation of motor outputs through somatosensory feedback. Although it is clear there is a spatial overlap of the sensory and motor areas in the brain, it is unclear how this affects motor control and performance.

In tasks requiring sub-maximal motor control, cutaneous sensory feedback is known to be critical as it provides haptic information during physical interactions with the external world. Removal of cutaneous feedback has been shown to increase errors in the performance of sub-maximal force production tasks requiring grasping and pinching (Nowak et al., 2001, 2004; Monzée et al., 2003). The production of sub-maximal forces has also been reported to be lower in magnitude and less accurate in patients who have lost cutaneous feedback due to sensory neuropathies (Forget and Lamarre, 1987; Cole and Sedgwick, 1992). There is limited knowledge, however, on the role cutaneous feedback plays in maximum motor output tasks. Typically, tasks requiring maximum motor output are considered as functions of voluntary muscle activation and muscle size and strength. However, evidence from recent studies on the hand suggests cutaneous feedback plays a major role in maximum finger force production tasks. In a recent study, maximum finger force production during a quasi-static maximal voluntary pressing task was reduced by 25% when tactile feedback was removed *via* ring block digital anesthesia to the digits of the hand (Shim et al., 2012). It is unclear, however, if this strong role of cutaneous feedback observed in the hand extends to whole-body maximum effort motor tasks as well. Previous studies conducted on sub-maximal whole-body gait movements used cooling as a means to reduce cutaneous feedback in the feet (Magnusson et al., 1990; Eils et al., 2002, 2004; Lowrey et al., 2013; Ferguson et al., 2017). Following the cooling of the soles of the feet, a reduction was seen in the afferent firing response to vibratory stimuli (Lowrey et al., 2013). It has also been demonstrated that the pressure distribution under the feet during locomotion is significantly altered and a cautious pattern of walking is evoked following the cooling of the soles of the feet (Eils et al., 2002, 2004). Although these studies have demonstrated decreases in performance on sub-maximal whole-body tasks following cooling to reduce cutaneous sensory feedback, a gap of knowledge remains in terms of maximum motor output tasks involving the whole body. Understanding the role cutaneous sensory feedback plays in these max-effort movements is critical in explosive movement performance including sprinting and jumping and in understanding the significance and mechanism of the spatial overlap of the sensory and motor areas in the brain.

Therefore, the purpose of the current study was to investigate the role of cutaneous sensory feedback in a maximum motor output task of a squat jump. We employed cooling of the foot soles as a means to reduce the sensory feedback to S1 during squat jumps to assess whole-body explosive force production (Markovic and Jaric, 2004; Bobbert and Casius, 2005; Van Hooren and Zolotarjova, 2017). Based on the substantial drop in force output in the absence of cutaneous feedback in maximum finger force production (Shim et al., 2012), we hypothesized that the reduction of the cutaneous feedback to S1 through cooling of the foot soles in the current study would reduce the S1-to-M1 projection and subsequently the maximal squat jump performance.

## Materials and Methods

### Subjects

Fifteen healthy young adults (20.9 ± 1.4 years, 10 females and 5 males, 173.3 ± 10.3 cm, 72.4 ± 14.9 kg) were recruited to participate in the study. An *a priori* power analysis indicated that approximately 13 participants were needed to detect differences in the variables of interest related to vertical squat jumps with effect sizes of at least *d* = 0.60 as statistical significance with *α* = 0.05 and *β* = 0.80 (G*Power 3.1, Kiel, Germany). All participants were free of injury, chronic mobility impairments, and had no history of major lower extremity injury requiring surgical intervention. Participants also had no history of Raynaud’s disease, peripheral neuropathy, or related nerve damage. Written informed consent was obtained from all participants before the commencement of testing. All study procedures were approved by the University of Maryland College Park Institutional Review Board and all experiments were performed following approved guidelines and regulations.

### Experimental Procedure

All participants performed a simple dynamic stretching regimen (Dalrymple et al., 2010), to warm-up the lower body for the jumping tasks required in this experiment. Dynamic stretches included knee tucks, leg swings to the opposite hand, slow butt-kicks and calf raises. Following the dynamic stretching warm-up, the investigator demonstrated the correct technique for performing the maximum vertical squat jump and provided verbal instruction to the participants. Practice jumps were performed by participants while the investigator provided verbal feedback based on visual inspection of the vertical ground reaction force (vGRF) during the jump to ensure the participants were using the proper technique and minimal countermovement in the squat jump. Ground reaction force data produced by participants during jumping were collected using two force plates (1,000 Hz, Kistler, Amherst, NY, USA) with one foot on each plate. The data were filtered using a second-order Butterworth filter with a 100 Hz cut-off frequency.

The maximum vertical squat jump testing procedure used was similar to that of a previous jump study (Thomas et al., 2015). Briefly, these procedures asked the participants to assume a starting position with their hands placed on their hips and squat to a self-selected depth with each foot on a force plate. The starting position was held for 3-s as the investigators provided a 3–2–1 countdown. The 3-s hold at the initial squat position was used to eliminate the involvement of the stretch-shortening cycle (Thomas et al., 2015).

**Figure 1:**
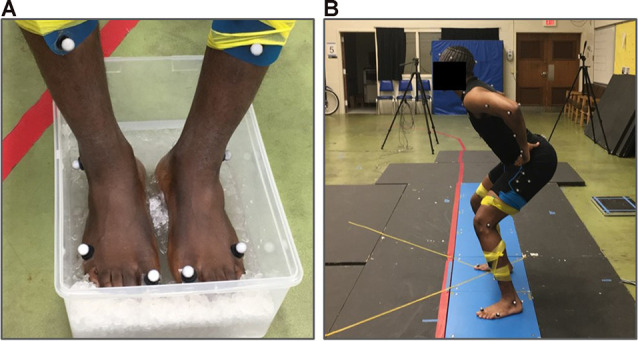
Experimental set-up. **(A)** An ice-water cooling bath used to cool the plantar surface of the feet for 15 min. **(B)** Example participant performing the squat jump task.

Participants performed the vertical squat jump under three different test conditions: baseline, cooled, and recovery. Three jump trials were performed under each of the three conditions. In the first condition, baseline maximum vertical squat jumps were performed. Subsequently, participants went through a cooling period which consisted of participants submerging only the plantar aspect of the feet in an ice-water bath maintained at approximately 0°C for 15 min (Figure 1A). In the second condition, jumps were performed immediately following the 15-min cooling period. The 15-min cooling period was selected to ensure the reduction of cutaneous sensory feedback based on previous studies which found that at least 10 min of cooling the foot soles was needed to reduce cutaneous receptor response to vibration to below 50% of baseline firing (Lowrey et al., 2013) and to see no additional reduction in sensation with tactile perception testing (Eils et al., 2002). Finally, the third condition was the recovery condition in which jumps were performed following 15-min of natural recovery from cooling. The equally timed 15-min cooling and recovery periods were used to provide equal amounts of time for both cooling and recovery across all subjects. Temperature measurements of the plantar aspect of the feet were taken immediately before the performance of each jump condition using an IR thermometer (Fluke 62 Max IR Thermometer, Fluke Corporation, Everett, WA, USA). All maximum vertical squat jumps were performed barefoot (Figure 1B).

### Data Analysis

**Figure 2:**
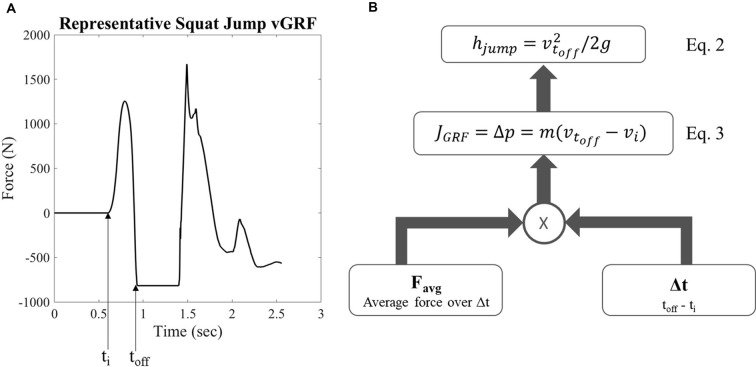
Representative vertical ground reaction force (vGRF) and calculation for jump height. **(A)** Representative vGRF for maximum vertical squat jump, *t*_i_ is the time of movement initiation and *t*_off_ is the time of takeoff **(B)** schematic of equations examining variables used to calculate jump height where *h*_jump_ is the maximum vertical jump height, *v*_off_ is the takeoff velocity, *g* is gravity, *J* is the impulse of the ground reaction force, *p* is momentum, *m* is mass, *v*_i_ is initial velocity, and *F*_avg_ is the average force produced during the moment initiation ground contact phase.

Jump height was calculated from the vGRF data using a custom MATLAB code (MathWorks, Natick, MA, USA). Body weight was first subtracted from the vGRF of each trial as shown by a representative sample in Figure 2A. The impulse of the ground contact phase during movement initiation for each trial was calculated using Equation 1.

(1) $$J=\int_{t_{\text{i}}}^{t_{\text{off}}}(F_{\text{vGRF}}-mg)dt=J_{\text{vGRF}}-J_{\text{BW}}=\Delta p=F_{\text{avg}}*\Delta t$$

where *J* is the impulse, *F*_avg_ is the average vGRF during the ground contact phase before takeoff, *t* is time, *m* is mass, *g* is gravity, and *p* is momentum. The impulse *J* was used to calculate the velocity at takeoff (Equation 3, Figure 2B), where *m* is the mass of the subject, *v*_toff_ is the takeoff velocity, and *J* is the impulse. The takeoff velocity was used with a standard kinematic equation to calculate the jump height (Equation 2, Figure 2B), where *h*_jump_ is maximum jump height (Figure 2B). The initiation of the jumping movement was determined as the point at which the vGRF reached three standard deviations above the mean of the static holding period before the jump.

### Statistical Analysis

Statistical analysis was performed using a customized script in R (Vienna, Austria). One-way repeated-measures ANOVA tests were conducted to compare the outcome variables of interest: impulse, average vGRF, the time duration of the ground contact phase during movement initiation, and maximum vertical squat jump height between the baseline, cooled, and recovery conditions. All data met the statistical assumption of normality (Shapiro–Wilk test) and Mauchly’s test revealed no violations of the sphericity assumption. *Post hoc* Tukey pairwise comparisons were used for multiple comparison contrasts. The level of statistical significance was set at *p* < 0.05. Cohen’s *d* effect sizes (ES) were calculated to determine the effect of the jump condition on the outcome variables. Effect sizes less than 0.2 were considered trivial, between 0.2 and 0.5 small, between 0.5 and 0.8 medium, and greater than 0.8 larger (Cohen, 1988). A table is included in the Supplementary Materials to provide the means and standard deviations of the outcome variables.

## Results

### Temperature

The mean skin surface temperature of the soles of the feet for the baseline, cooled, and recovered conditions was 24.91°C (SD = 1.96), 11.85°C (SD = 2.64), and 22.26°C (SD = 2.79), respectively. There was a significant difference for the foot sole temperature between all conditions (*p* < 0.001).

### Maximum Vertical Squat Jump Performance

**Figure 3:**
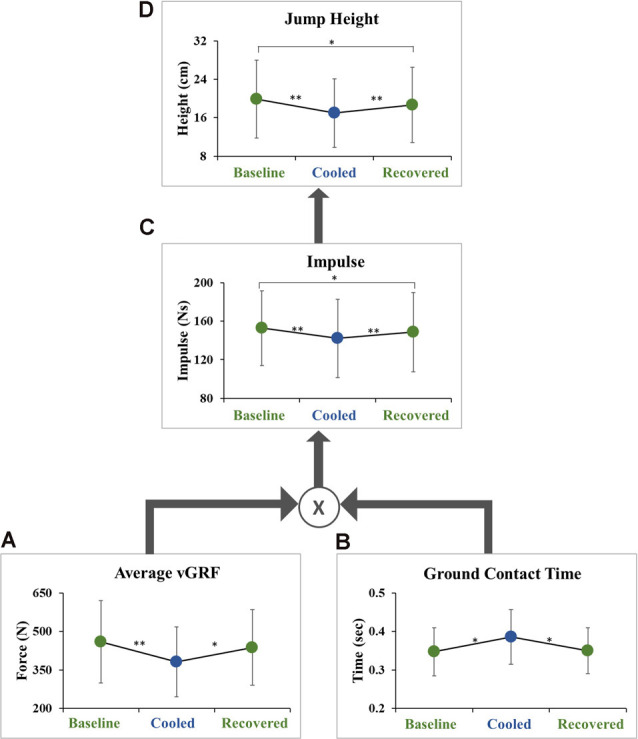
Hierarchical schematic of jump performance outcome variables. **(A)** The average vGRF produced during the ground contact time during movement initiation, **(B)** ground contact time during movement initiation, **(C)** impulse during the movement initiation phase, and **(D)** maximum jump height. Each display mean (±SD) for baseline, cooled and recovered conditions. *p < 0.05, **p < 0.001.

Jump height was lower in the cooled condition compared to the baseline condition (18.5 ± 5.6 cm vs. 21.6 ± 6.2 cm, *p* = 3.39 × 10^−8^, ES = 0.52) and to the recovered condition (20.4 ± 6.2 cm, *p* = 0.00124, ES = 0.30). There was also a significant difference between the baseline and recovered conditions (*p* = 0.022, ES = 0.21; Figure 3D). The data were further analyzed to examine vGRF impulse, average vGRF, and ground contact time before takeoff (Figure 3). Pre-takeoff vGRF impulse was lower in the cooled condition compared to the baseline condition (142.1 ± 40.7 Ns vs. 152.7 ± 38.9 Ns, *p* = 2.16 × 10^−8^, ES = 0.26) and the recovered condition (148.5 ± 41.2 Ns, *p* = 0.000672, ES = 0.16). A significant difference in vGRF impulse was seen between the baseline and recovered conditions (*p* = 0.03, ES = 0.10; Figure 3C). The average vGRF and contact time pre-takeoff were also examined as these two components can be used to calculate impulse (Equation 1). Average vGRF before takeoff was lower in the cooled condition compared to the baseline condition (380.9 ± 136.3 N vs. 459.1 ± 160.8 N, *p* = 8.1 × 10^−5^, ES = 0.52) and the recovered condition (437.6 ± 147.5 N, *p* = 0.0043, ES = 0.40). The average vGRF impulse before takeoff, during the movement initiation phase, was not significantly different between the baseline and recovered conditions (*p* = 0.2774, ES = 0.14; Figure 3A). The ground contact time during movement initiation before takeoff was greater in the cooled condition compared to the baseline (0.39 ± 0.07 s vs. 0.35 ± 0.06 s, *p* = 0.014, ES = 0.58) and the recovered condition (0.35 ± 0.05 s, *p* = 0.022, ES = 0.55). There was no significant difference between the baseline and recovered condition (*p* = 0.865, ES = 0.04; Figure 3B).

## Discussion

This study examined the performance of a maximal motor output task in the form of a squat jump under baseline, cooled, and recovered conditions. To our knowledge, this is the first study to investigate the effects of the reduction of cutaneous feedback on maximal motor outputs in a whole-body human movement. Previous studies using anesthesia for eliminating cutaneous feedback have used quasi-static paradigms (Nowak et al., 2001, 2004; Monzée et al., 2003), while others have investigated the influence of entire lower limb muscle cooling on maximal muscle strength and dynamic whole-body movements (Bergh and Ekblom, 1979). The cooling of small muscles inside the feet might have negatively influenced the jump performance. However, we expect that the influence was relatively minimal as the jumping is performed mainly by the large muscles in the plantar flexors, knee extensors, and hip extensors. The present study is the first to examine a dynamic maximal output whole-body human movement focused on the reduction of cutaneous sensory feedback.

Our hypothesis that the squat jump height would decrease following the cooling of the plantar surfaces of the feet was supported. The decrease in squat jump height following reduction of cutaneous sensory feedback *via* cooling is consistent with findings from previous studies on maximal finger force production and studies of gait (Eils et al., 2002, 2004; Shim et al., 2012). In other movements/tasks, removal of cutaneous feedback reduced maximum force during finger pressing tasks using the four-finger digits together and separately by about 25% (Shim et al., 2012) and altered gait patterns towards more cautious ground contact in the sub-maximal task of walking as indicated by a delayed first ground contact peak and significantly reduced braking force peak in the iced condition (Eils et al., 2004). The decrease in the average vGRF produced before takeoff immediately following cooling in the present study is consistent with the decreases seen in pressure distribution during walking immediately following cooling (Eils et al., 2002).

The increase in ground contact time may indicate a strategy used to compensate for the reduction of cutaneous feedback. No difference in contact time was seen following reduction of cutaneous sensation *via* cooling in walking tasks (Eils et al., 2002), which indicates the increase in ground contact time duration may be unique to tasks requiring maximal motor output such as jumping as compared to the sub-maximal task of walking. Vertical impulse (average vGRF multiplied by contact time) dictates the jump height, and participants responded to the evident loss of maximum force production by increasing contact time duration. However, this was not enough to achieve the impulse needed to match the baseline squat jump height, indicating significant effects of the induced cooling and reduction of sensory feedback.

The results of the present study therefore demonstrate the importance of sensory information in the performance of maximal motor output tasks. The decrease in squat jump height, compared to the baseline and recovered conditions suggests that when performing this type of action the CNS is dependent on sensory information in addition to motor information, which may be due to the neuroanatomical connections between the S1 and M1 areas in the brain. Recent studies indicate that there is an overflow of information from S1 into M1 in rat and mice models (Matyas et al., 2010; Petrof et al., 2015). The current findings indicate this overflow may also be occurring in humans. However, further research is required to understand the pathways and exact mechanisms behind the sharing of information between S1 and M1 in humans. Moreover, although no known mechanism can directly explain the changes found in maximal vertical squat jump performance due to the reduction of sensory feedback *via* cooling in terms of subcortical neural connection and interneurons at the spinal level, possibly these could be contributors to the findings as well. Finally, psychological and cognitive factors related to cooling have been shown to influence motor performance with prolonged cooling exposure (Bensel and Lockhart, 1974; Enander, 1987; O’Brien et al., 2007). In the case of the present study, however, since the cooling time was only 15 min and only the plantar cutaneous surface of the feet was targeted for reduction of sensory feedback, it is less likely that these psychological factors played a role in our study.

It is well understood that maximal vertical squat jump performance is constrained by the properties of the musculoskeletal system (Bobbert, 2001; Nagano and Gerritsen, 2001). However, the findings of the current study demonstrate the need to consider the contributions of somatosensory information along with the constraints imposed by the musculoskeletal system. In the present study, only cutaneous sensory feedback was reduced leaving all other properties of the musculoskeletal system unchanged. This manipulation demonstrated significant differences in the average vGRF production, contact time, and impulse of the jump preparation phase as well as the squat jump height.

Reducing cutaneous sensory feedback in the feet *via* cooling is limited as compared to a complete anesthesia block used in many finger studies (Augurelle et al., 2003; Reilly et al., 2008; Shim et al., 2012). Though not as complete as an anesthetic block, the results of the current study demonstrate significant findings in the cooled squat jump condition as compared with the baseline and recovered conditions indicating that the reduction of cutaneous feedback *via* cooling is an effective method in the case of the task of a squat jump. One limitation of this study is in the use of equal lengths of cooling and recovery time. Though this timing allowed for consistency across participants, the length of the recovery period was not, in all cases, long enough for the temperature of the soles of the feet to return to baseline temperature. Furthermore, this study was limited in its use of foot sole temperature measurements rather than a direct measurement of sensory feedback reduction. Future studies may consider making the period for natural rewarming of the feet unique to each subject to ensure the temperature returns back to the original baseline temperature as well as using a direct measure of sensory feedback. Additional future studies should use fMRI to directly examine the neural mechanisms between S1 and M1 during a maximal force production task such as an isometric finger pressing or an isometric ankle plantarflexion task.

In conclusion, this study reports decreases in squat jump height and average vGRF during squat jumping after the reduction of cutaneous sensory feedback *via* cooling of the plantar surface of the feet. Though the neuromechanical mechanisms for this finding require further investigation, these findings suggest that cutaneous sensory feedback plays a critical role in explosive movements in humans.

## Data Availability Statement

The raw data supporting the conclusions of this article will be made available by the authors, without undue reservation.

## Ethics Statement

The studies involving human participants were reviewed and approved by University of Maryland College Park IRB. The patients/participants provided their written informed consent to participate in this study.

## Author Contributions

MC and JS conceived and designed the research experiment. MC and JS performed the experiments. MC, GG, and JS analyzed the data. MC, HK, RM, and JS wrote the manuscript. MC, GG, HK, RM, and JS all reviewed the manuscript. All authors contributed to the article and approved the submitted version.

## Conflict of Interest

The authors declare that the research was conducted in the absence of any commercial or financial relationships that could be construed as a potential conflict of interest. The reviewer YK declared a past co-authorship with one of the authors JS to the handling editor.
