# Supplementary material for: Sensory-to-Motor Overflow: Cooling Foot Soles Impedes Squat Jump Performance
Source: Front Hum Neurosci. 2020 Oct 9;14:549880. doi: 10.3389/fnhum.2020.549880 (PMC7581857; doi:10.3389/fnhum.2020.549880)
Supplement: Supplementary file 1 [file Data_Sheet_1.PDF]

Supplementary Material

Table 1. Mean ± SD for outcome variables: jump height, impulse, average vGRF, and ground contact time.

	Baseline	Cooled	Recovered
Jump Height (cm)	19.9 ± 8.1	17.0 ± 7.1	18.6 ± 7.8
Impulse (Ns)	152.7 ± 38.9	142.1 ± 40.7	148.5 ± 41.2
Average vGRF (N)	459.1 ± 160.8	380.9 ± 136.3	437.6 ± 147.5
Ground Contact Time (s)	0.35 ± 0.06	0.39 ± 0.07	0.35 ± 0.06
